# MULTIPLE CALLUSES AS OCCUPATIONAL MARKS IN SLIPPER-STRAP MAKERS

**DOI:** 10.4103/0019-5154.44797

**Published:** 2008

**Authors:** T P Vetrichevvel, R Sureshbabu, C Udayashankar, P Oudeacoumar

**Affiliations:** *From the Department of Dermatology and STD, Aarupadai Veedu Medical College and Hospital (AVMC and H), Pondicherry – 607 402, India*

Sir,

While inspecting the hands of the mother of a child with scabies, we had noticed multiple calluses on the fingers of her right hand. One of them was located over the dorsolateral surface of middle phalanx of the middle finger and was of size 1.2 × 0.9 cm (Fig. 1). The other callus was located on the dorsomedial surface of the ring finger measuring 0.8 × 0.7 cm. She also had a curvilinear band of thickening on the palmar aspect of the metacarpophalangeal joint of her right thumb culminating in a callus of size 1.2 × 1 cm on the lateral side (Fig. 2). The lesions had been present for nearly 6 years, initially small, increasing in size over the years. There was no history of fluid or pus-filled lesions, contact with cattle or similar lesions on the fingers of her left hand or toes. She, along with many of the women folk in her hamlet is engaged in making straps for slippers, with each one of them producing on an average 250 pairs of straps per day. The special scissors used for this purpose has two round “eyes,” and it is held with the base of the thumb placed in one eye, while both the middle and ring finger are placed in the other (Fig. 3). This enables them to exert adequate pressure to cut through the rubber mould. Although varying in size, calluses at the same sites were noted in all the professionals we had seen during the house visit. The calluses are asymptomatic, but for their cosmetic appearance.

**Fig. 1:**
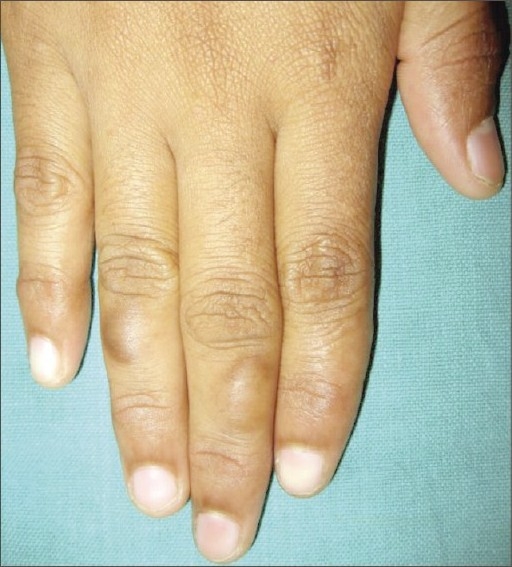
Calluses on the dorsum of middle and ring finger

**Fig. 2:**
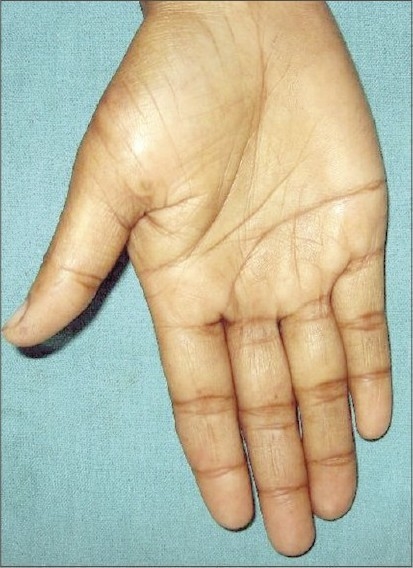
Curvilinear band of thickening at the base of thumb

**Fig. 3:**
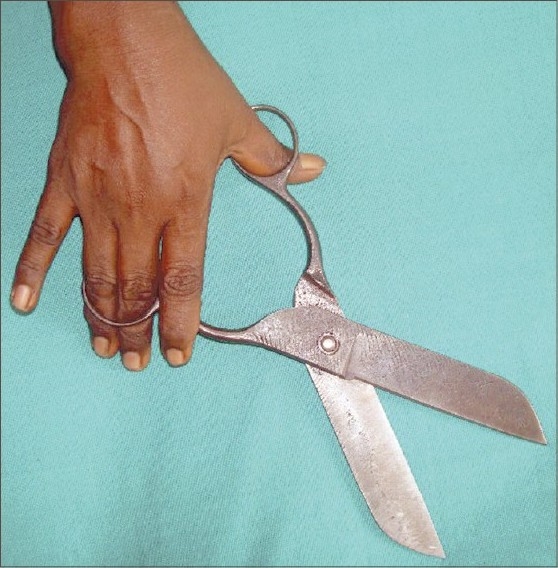
Scissors, as held by slipper-strap makers

Scissors-induced calluses have a pattern that almost defines the occupation. They are bound to occur in hairdressers, tailors/dressmakers and gardeners, but have also been reported to occur with beedi rollers and glove makers.^1^,^2^ The site of occurrence of the callus depends on how the scissors are held, while its size depends on the frequency and duration of usage. The morphology of the calluses also depend on the size and weight of the scissors used, the target to be cut and the techniques used for prevention or treatment, including self paring.

Hairdressers use scissors with two small round eyes^3^ held by the ring finger and thumb and hence develop calluses predominantly on the medial and posterior surface of middle of the ring finger and on the medial and lateral surface of the base of the thumb of the dominant hand.

Scissors used by tailors and dress makers are usually heavy and have one oval and one round eye.^3^ The round eye is held by the thumb, while all the other four fingers are placed inside the oval eye for good maneuverability. This explains the occurrence of calluses on the lateral portion of the base of the thumb, lateral aspect of the lower portion of the index finger and middle of the medial portion of the little finger consistently as they are the sites of maximum contact and friction. On prolonged usage, calluses can also form on knuckles of other fingers of the dominant hand.^1^

Beedi rollers also use scissors with two round but larger eyes and have been reported to have calluses on the sides and dorsa of fingers, especially right middle and index fingers.^2^ Gardeners use a variant of scissors called shears and can develop calluses on the palms. Glove-makers use knives in addition to scissors and develop callosities and deformities of hands and fingers.^1^

Calluses in slipper-strap makers are unique in a way that there are two well-defined calluses on two adjacent fingers, the middle and ring finger and on the thumb, a pattern caused by the specific way they hold the scissors and cannot be attributed to any other profession. Also, these calluses start appearing earlier, within 1 or 2 months and are related to the pressure against a firmer target.

Use of rubber cushioning at the rims of the eyes of the scissors, use of plastic or lightweight scissors, regular sharpening of scissors and lesser work timings are some prevention methods. Regular use of emollients and keratolytics are treatment options.^4^ With increasing automation and mechanization, these occupational marks are bound to decrease, but identification of such occupational trauma and appropriate healthcare advice will help decrease the incidence of these calluses, avoid formation of large disabling calluses, prevent complications like fissuring or infection and improve work efficiency.

## References

[1] Kanerva L, Kanerva L, Elsner P, Wahlberg JE, Maibach HI (2000). Occupational marks. Handbook of occupational dermatology.

[2] Kuruvila M, Mukhi SV, Kumar P, Rao GS, Sridhar KS, Kotian MS (2002). Occupational dermatoses in Beedi rollers. Indian J Dermatol Venereol Leprol.

[3] Facts about scissors. http://www.canadacutlery.com/product/facts_on_scissors.pdf.

[4] Taylor JS, Sood A, Freedberg IM, Eisen AZ, Wolff K, Austen KF, Goldsmith LA, Latz SI (2003). Occupational skin disease. Fitzpatrick's dermatology in general medicine.

